# Supplementary material for: Stargardt-like Clinical Characteristics and Disease Course Associated with Variants in the WDR19 Gene
Source: Genes (Basel). 2023 Jan 22;14(2):291. doi: 10.3390/genes14020291 (PMC9957452; doi:10.3390/genes14020291)
Supplement: Supplementary file 1 [file genes-14-00291-s001.zip › genes-2122455-supplementary.pdf]

Table S1. Reported pathogenic and likely pathogenic variants in the *WDR19* gene, including variants found in our patient, and associated phenotypes. As a RefSeq NM_025132.4 canonical transcript was used in GRCh37 (hg19) assembly.

Exon/ Intron	DNA change	Protein	Clinical classification	ClinVar ID	rsID (dbSNP)	Reference	Reported/ ClinVar phenotype	Domain
29	c.(3183+1_3184-1)_(3261+1_3262-1)del	p.(?)	variant of unknown significance	N/A	N/A	Our patient	Stargardt-like disease	
14	c.1454G>T	p.(Ser485Ile)	variant of unknown significance	N/A	N/A	PMID: 28559085, Our patient	Stargardt-like disease (1x),	
11	c.1031A>G	p.(His344Arg)	likely pathogenic	N/A	rs376527958	PMID: 28559085	Stargardt-like disease (1x)	WD 6
14	c.1434C>G	p.(Ile478Met)	pathogenic/ likely pathogenic	266105	rs886039814	PMID: 27894351	Senior-Loken (2x) Cranioectodermal dysplasia (1x)	
32i	c.3565+1G>A	p.?	pathogenic	127159	rs587777352	PMID: 22019273, 23559409, 26489029, 29068549, 23683095	Nephronophthisis (1x), Senior-Loken (2x), Asphyxiating thoracic dystrophy (2x)	
6	c.475G>A	p.(Asp159Asn)	pathogenic	446637	rs1451698951	PMID: 29068549	Short-rib polydactyly syndrome (2x)	WD 4
9	c.746T>C	p.(Phe249Ser)	likely pathogenic	446640	rs1553905326	PMID: 29068549	Asphyxiating thoracic dystrophy (2x)	
9	c.781dup	p.(Thr261Asnfs*13)	pathogenic	446634	rs748656635	PMID: 29068549, 23559409, 23683095	Nephronophthisis / Senior-Loken (1x), WDR19-related disorder (1x), Asphyxiating thoracic dystrophy (3x)	
9	c.817A>G	p.(Asn273Asp)	pathogenic / variant of unknown significance	446633	rs375644378	PMID: 29068549	Asphyxiating thoracic dystrophy (3x)	WD 5
9	c.880G>A	p.(Gly294Arg)	likely pathogenic	446635	rs377160857	PMID: 29068549	Asphyxiating thoracic dystrophy (3x)	WD 5
23	c.2563C>T	p.(Gln855*)	pathogenic/ likely pathogenic	446642	rs772599282	PMID: 29068549	Asphyxiating thoracic dystrophy (2x)	TPR3

23	c.2585T>C	p.(Leu862Pro)	likely pathogenic/ variant of unknown significance	N/A	rs1414268971	PMID: 29620725	Ciliopathies with major skeletal involvment	TPR3
28i	c.3184-2A>C	p.?	pathogenic	1473602	rs1020915921	PMID: 23683095, 29068549, 23559409, 22019273,	Senior-Loken syndrome 8/ Asphyxiating thoracic dystrophy	
31i	c.3484-2A>C	p.?	pathogenic	446636	rs1553918403	PMID: 29068549	Asphyxiating thoracic dystrophy (1x), Short-rib polydactyly syndrome (2x)	
33i	c.3716+1G>A	p.?	pathogenic	446639	rs1191056931	PMID: 29068549	Asphyxiating thoracic dystrophy (2x)	
34	c.3800G>A	p.(Cys1267Tyr)	pathogenic	446638	rs745603321	PMID: 29068549	Asphyxiating thoracic dystrophy (2x)	
1i	c.6+1G>T	p.?	pathogenic	N/A	N/A	PMID: 33323469	Nephronophtisis related ciliopathy	
2	c.31_35del	p.(Thr11Alafs*23)	pathogenic	N/A	N/A	VKGL data sharing initiative Nederland	N/A	WD 1
5i	c.407-2A>G	p.?	pathogenic	189380	rs374400438	PMID: 23683095	Senior-Loken syndrome 8/ retinitis pigmentosa	
8	c.641dup	p.(Leu214Phefs*5)	pathogenic	127154	rs587777348	PMID: 23683095, 23559409	Senior-Loken syndrome 8 (2x)	
8	c.641T>A	p.(Leu214Ter)	pathogenic/ likely pathogenic	632439	rs751290509	PMID: 22019273, 23559409, 23683095, 26275793, 27241786, 29068549	Cranioectodermal dysplasia (1x), Senior-Loken syndrome / Asphyxiating thoracic dystrophy (1x), Retinal dystrophy (1x), WDR19-related disorders (1x)	
8	c.682C>T	p.(Gln228*)	pathogenic	127156	rs587777350	PMID: 23559409	Nephronophtisis related ciliopathy	
14	c.1477G>C	p.(Asp493His)	pathogenic	127155	rs587777349	PMID: 23683095	Senior-Loken syndrome / Asphyxiating thoracic dystrophy (1x), Senior-Loken (1x), Nephronophtisis (1x)	
17i	c.1983-2A>T	p.?	likely pathogenic	N/A	N/A	PMID: 31725169	Retinitis pigmentosa (1x)	

25	c.2782A>T	p.(Ile928Phe)	pathogenic / variant of unknown significance	348744	rs780963454	PMID: 31725169	Asphyxiating thoracic dystrophy (2x), Cranioectodermal dysplasia (1x), Retinitis pigmentosa (1x)	TPR4
33	c.3703G>A	p.(Glu1235Lys)	pathogenic	127157	rs587777351	PMID: 33532864, 23559409, 31101064	Senior-Loken syndrome/ Asphyxiating thoracic dystrophy (1x), Senior-Loken syndrome (1x), Nephronophtisis (2x),	
18	c.2129T>C	p.(Leu710Ser)	likely pathogenic, variant of unknown significance	30703	rs387906980	PMID: 23683095, 22019273, 27241786, PMID: 28559086	Senior-Loken syndrome (2x), Senior-Loken syndrome/ Asphyxiating thoracic dystrophy (1x), Cranioectodermal dysplasia (1x), Syndromic early childhood onset retinal dystrophy (1x), arRP (1x)	
10-13	ex10-13del	p.?	likely pathogenic	N/A	N/A	PMID:31101064	Nephronophthisis (1x)	
25	c.2777G>T	p.(Ser926Ile)	likely pathogenic, pathogenic	191190	rs751386429	PMID: 29620724, 32055034	Cranioectodermal dysplasia (1x), Stargardt-like disease (3x)	TPR4
32	c.3533G>A	p.(Arg1178Gln)	likely pathogenic/ pathogenic, variant of unknown significance	127158	rs79436363	PMID: 23559409	Nephronophtisis (1x), Senior-Loken syndrome (2x), Leber congenital amaurosis (1x), Cranioectodermal dysplasia (1x), Senior-Loken syndrome/ Asphyxiating thoracic dystrophy (1x)	
15	c.1483G>C	p.(Gly495Arg)	pathogenic/ likely pathogenic	446641	rs1215108056	PMID: 29068549	Asphyxiating thoracic dystrophy (3x)	
9	c.812C>T	p.(Ala271Val)	likely pathogenic, Variant of unknown significance	1514580	rs981943939	N/A	Asphyxiating thoracic dystrophy (1x)	
22	c.2561A>C	p.(Lys854Thr)	likely pathogenic	N/A	N/A	PMID: 29068549	Asphyxiating thoracic dystrophy (1x)	TPR3
2	c.20T>C	p.(Leu7Pro)	likely pathogenic	30705	rs387906982	PMID: 22019273	Short-rib thoracic dysplasia without polydactyly syndrome (1x)	

2	c.89_90insTACT	p.(Val31Thrfs*6)	likely pathogenic	N/A	N/A	PMID: 32037395	Retinal dystrophy	WD 1
11	c.1034T>G	p.(Val345Gly)	likely pathogenic	30706	rs387906983	PMID: 22019273	Nephronophtisis (1x)	WD 6
23i	c.2645+1G>T	p.(?)	likely pathogenic	1686924	N/A	PMID: 33946315, 28621010	Cranioectodermal dysplasia (1x)	
25	c.2777G>T	p.(Ser926Ile)	likely pathogenic	191190	rs751386429	PMID: 26355662	arRP (1x)	TPR4
27	c.3068dup	p.(Tyr1023*)	likely pathogenic	30707	rs786205114	PMID: 22019273	Nephronophtisis (1x)	TPR6
27	c.3066_3067del	p.(Tyr1023Serfs*18)	likely pathogenic	N/A	N/A	PMID: 28559085	Syndromic early childhood onset retinal dystrophy (1x)	TPR6
30	c.3307C>T	p.(Arg1103*)	likely pathogenic	30704	rs387906981	PMID: 22019273, 24027799	Senior-Loken syndrome/ Asphyxiating thoracic dystrophy (1x), Cranioectodermal dysplasia (2x)	
10-14	NC_000004.11:g.(?_39215680)_(39219295_?)del	p.(?)rRP (1x)	pathogenic	625691	N/A	N/A	Nephronophtisis (1x)	WD5-WD6
22-28	NC_000004.11:g.(?_39245868)_(39259174_?)del	p.(?)	variant of unknown significance	1042683	N/A	N/A	Senior-Loken syndrome/ Asphyxiating thoracic dystrophy (1x)	
27	NC_000004.11:g.(?_39257448)_(39257600_?)del	p.(?)	pathogenic	1457671	N/A	N/A	Senior-Loken syndrome/ Asphyxiating thoracic dystrophy (1x)	

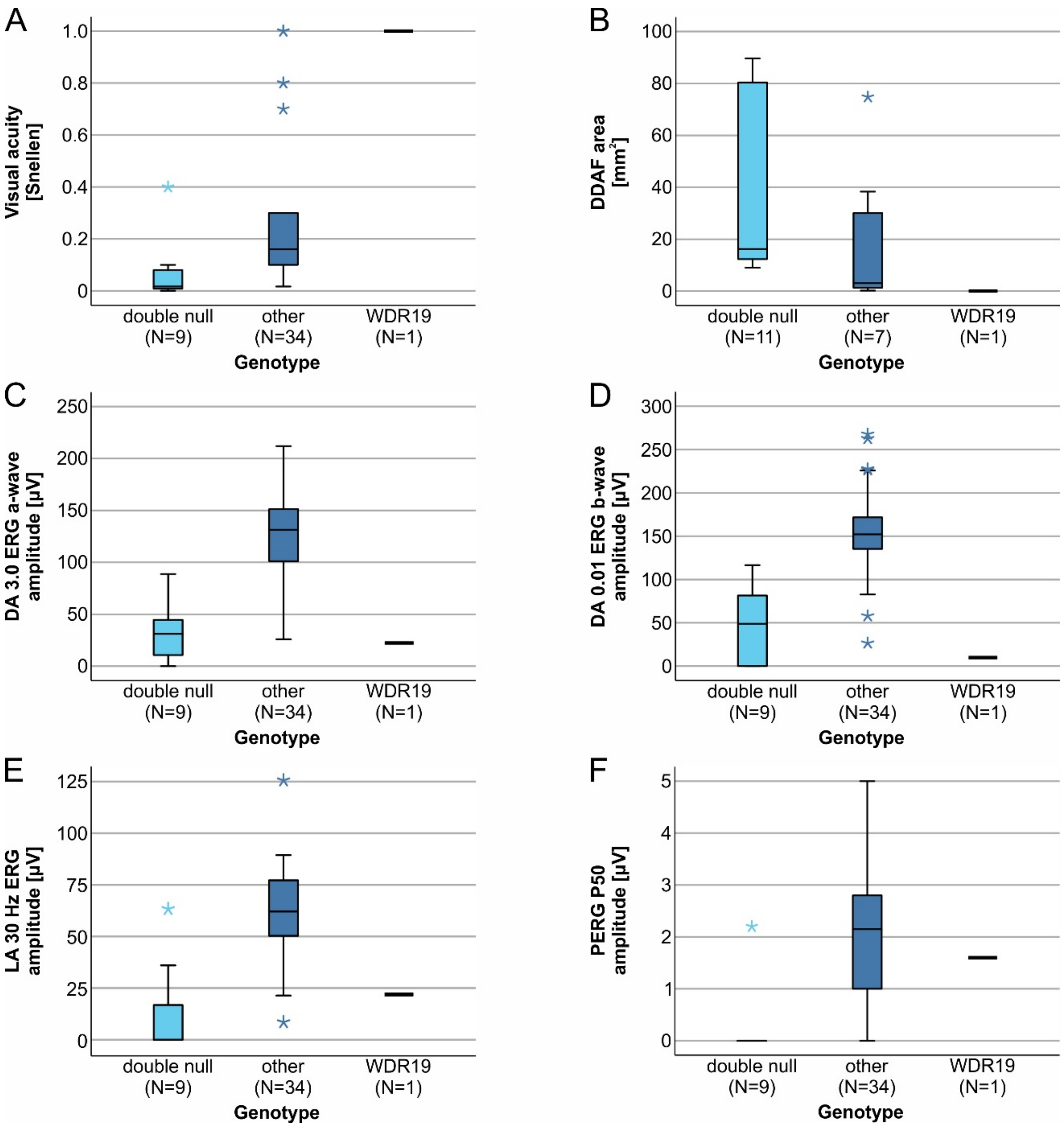

Figure S1. Boxplot charts showing measured parameters in patients with different genotypes. Horizontal lines represent median values, boxes half of the data and whiskers the remaining data except in the case of the outliers (stars). VA—visual acuity, DDAF—definitely decreased autofuorescence, ERG—electroretinography, dark-adapted, DA—dark-adapted, LA—light-adapted, PERG—pattern ERG.
